# Gene silencing of cathepsins B and L using CTV-based, plant-mediated RNAi interferes with ovarial development in Asian citrus psyllid (ACP), *Diaphorina citri*

**DOI:** 10.3389/fpls.2023.1219319

**Published:** 2023-09-29

**Authors:** Freddy Ibanez, Sâmara Vieira Rocha, William O. Dawson, Choaa El-Mohtar, Cecile Robertson, Lukasz L. Stelinski, Andrea Soares-Costa

**Affiliations:** ^1^ Department of Entomology, Texas A&M AgriLife Research, Weslaco, TX, ;United States; ^2^ Department of Genetics and Evolution, Federal University of São Carlos, São Carlos, SP, ;Brazil; ^3^ Plant Pathology Department, Citrus Research and Education Center, University of Florida, Lake Alfred, FL, ;United States; ^4^ Department of Entomology and Nematology, Citrus Research and Education Center, University of Florida, Lake Alfred, FL, ;United States

**Keywords:** Diaphorina citri, Huanglongbing, CTV, RNAi, Cathepsin B, Cathepsin L

## Abstract

*Diaphorina citri* Kuwayama (Hemiptera: Liviidae) is a vector of the bacteria *Candidatus* Liberibacter americanus (*C*Lam) and *Candidatus* Liberibacter asiaticus (*C*Las), which are phloem-restricted and associated with the most important and destructive worldwide citrus disease, Huanglongbing (HLB). Currently, no cure for HLB has been described. Therefore, measures have focused on reducing *D. citri* populations. In these insects, cathepsin B (DCcathB) and L (DCcathL) enzymes play an important role in digestion, and are involved in embryogenesis, immune defense, and ecdysis. In this study, we used a CTV-based vector to deliver dsRNA (CTV-dsRNA) into *Citrus macrophylla* plants targeting *DCcathB* and *DCcathL* genes in *D. citri* that fed on the phloem of these CTV-RNAi infected plants. Subsequently, we evaluated expression of *DCcathB* and *DCcathL* genes as well as the *Vitellogenin* (*Vg)* gene by RT-qPCR in *D. citri* fed on CTV-dsRNA occurring in plant phloem. It was found that a defective phenotype in *D. citri* females as a result of knockdown of *DCcathB* and *DCcathL* genes mediated by CTV dsRNA. These results showed that Psyllids fed on plants treated with the CTV-dsRNA exhibited downregulation of the Vg gene, one of the most important genes associated with embryogenic and female development, which was associated with dsRNA-mediated silencing of the two cathepsin genes. Based on our findings, a CTV-based strategy for delivering RNAi via plants that targets *DCcathB* and *DCcathL* genes may represent a suitable avenue for development of dsRNA-based tools to manage *D. citri* that limits the spread of HLB.

## 1 Introduction

The Asian Citrus Psyllid (ACP), *Diaphorina citri* Kuwayama (Hemiptera: Liviidae) is a vector of the phytopathogens *Candidatus* Liberibacter americanus (*C*lam) (Coletta-Filho et al., 2005; Teixeira et al., 2005a; Teixeira et al., 2005b; Teixeira et al., 2005c) and *Candidatus* Liberibacter asiaticus (*C*Las) (Garnier and Bové, 1996; Bové, 2006). Both are phloem-restricted, belong to the alpha-proteobacteria group (Jagoueix et al., 1994), and are associated with the most serious citrus disease in the world, Huanglongbing (HLB) (McClean and Schwartz, 1970; Bové, 2006). The symptoms of HLB are well characterized, including reduced vigor (Etxeberria et al., 2009), a decline in fruit production, reduced fruit size and quality, aborted seeds, fruit asymmetry, an increase in juice acidity and, in more severe stages, death of the trees (Bové, 2006; Etxeberria et al., 2009) resulting in a significant economic loss. The life cycle of the *D. citri* vector includes an egg stage, 5 nymphal instars, and an adult stage; both immature and adult stages are classified as phloem feeding and sap-sucking insects and the duration of each developmental stage is dependent of the host-species and temperature (Tsai and Liu, 2000). *D. citri* population abundance is closely linked to the occurrence of young citrus leaf flush, which is the only suitable site for egg laying and nymph development (Hall and Albrigo, 2007). Given the high osmotic pressure and sugar content present in plant phloem-sap, hemipterans possess sucrose-transglucosidases in the alimentary canal that allow them to tolerate and transform the excessive sugars ingested into long chains of oligosaccharides that are subsequently excreted as honeydew (Douglas, 2006).

Currently, there is no known cure for HLB, and most measures associated with management of the disease focus on reducing *D. citri* populations. Over the past decade, most *D. citri* management programs have heavily relied on insecticide sprays (Qureshi et al., 2014; Boina and Bloomquist, 2011). However, this has resulted in significantly higher production costs, and unintended ecological consequences, such as decreased populations of beneficial insects and development of insecticide resistance among *D. citri* populations (Tiwari et al., 2011; Farnsworthn et al., 2014). Therefore, alternatives to insecticides must be developed and integrated into vector suppression.

One strategy that has received significant attention in recent years as a possible alternative to traditional insecticides is RNA interference (RNAi) (Huvenne and Smagghe, 2010; Mamta and Rajam, 2017; Yan et al., 2020). The purpose of RNAi is to silence specifically targeted genes involved in critical biological processes in the pest, but which are sufficiently specific to minimize non-target consequences. For example, knockdown of *arginine kinase, abnormal wing disc, carboxylesterases, muscle protein 20, cathepsin D, chitin synthase* and *sucrose hydrolase* genes cause various physical deformities and mortality in *D. citri* and could theoretically reduce *D. citri* populations (El-Shesheny et al., 2013; Hajeri et al., 2014; Andrade and Hunter, 2017; Galdeano et al., 2017; Kishk et al., 2017; Yu et al., 2017; Santos-Ortega and Killiny, 2018). The RNAi mechanism is triggered by a double-stranded RNA molecule (dsRNA) precursor, of variable length and origin, which is excised by RNase-III-like endonuclease (Dicer) into short ~20-30 nucleotides RNAs duplexes, called short interfering RNAs (siRNAs). The siRNAs are recognized by the RNA-induced silencing complex (RISC) and then this complex recognizes the expected target mRNA (single-strand), cleaving the mRNA molecule and thereby hindering the normal translational process (Elbashir et al., 2001; Hammond et al., 2001; Meister and Tuschl, 2004; Whyard et al., 2009). In agricultural systems, the potential implementation of RNAi delivery is based on transgenic and non-transgenic strategies. Among non-transgenic strategies to deliver RNAi, the use of Virus-induced gene silencing (VIGS) has been explored (Bachan and Dinesh-Kumar, 2012; Wuriyanghan and Falk, 2013; Hajeri et al., 2014; Khan et al., 2015). This strategy uses an engineered plant virus to deliver a targeted insect gene-sequence (dsRNA/siRNAs) during virus replication and/or transcription in the plant hosts (Bachan and Dinesh-Kumar, 2012). Upon feeding on a host colonized with the virus vector delivering the dsRNA/siRNA, the target pest ingests them leading to an inhibition of translation of the targeted genes within the insect (Hajeri et al., 2014; Khan et al., 2018).

In citrus, virus-based vectors have been created through the manipulation of *Citrus tristeza virus* (CTV) for heterologous gene expression (Folimonov et al., 2007; El-Mohtar and Dawson, 2014). CTV is a member of the Closterovirus genus within the family Closteroviridae; it possesses a single stranded RNA genome of ~19.3 kb (Bar-Joseph et al., 1979; Karasev et al., 1995), and it is considered the largest and most complex plant RNA virus with phloem limited tissue tropism where ACP feeds. The CTV-RNAi vectors were successfully used to target citrus and ACP genes (Hajeri et al., 2014). For example, engineering the CTV virus vector to deliver a 400 base, truncated RNA has been successfully demonstrated in citrus, by targeting the phytoene desaturase (PDS) gene, which resulted in photo-bleaching of citrus leaves upon infection with the CTV vector. Also, a CTV-RNAi vector delivering a truncated version of the *Awd* gene of *D. citri* resulted in reduced expression of the Awd gene upon ACP feeding on infected plants causing malformation of *D. citri* wings and high mortality of adults (Hajeri et al., 2014). This validates the use of CTV vectors for delivery of dsRNAs/siRNAs via citrus to target *D. citri* genes.

In our previous studies, two cathepsin genes were identified in *D. citri*, *cathepsin B-like* (DcCathB) (Ferrara et al., 2015) and *cathepsin L-like* (DcCathL) (Ferrara et al., 2020). Cathepsins are proteolytic cysteine-peptidase type enzymes (Storer and Ménard, 1994; Polgár, 2013; Rawlings et al., 2016), belonging to the family C1 and subfamily papain; cathepsins are proteases that occur broadly among animals and play important roles in immunity, metabolism, and development (Sajid and McKerrow, 2002; Wiederanders, 2003). In insects, cathepsins B and L play a key role in digestion and are involved in embryogenesis, immune defense, and ecdysis (Terra and Ferreira, 1994). In *D. citri*, DcCathB and DcCathL were highly expressed in the alimentary canal suggesting a role in digestion and were expressed during the egg stage suggesting involvement in embryonic development (Ferrara et al., 2020). We hypothesized that blocking activity of these enzymes using the recombinant CTV-RNAi vectors should disturb critical biological processes in *D. citri*. In this study, we evaluated the effects of RNAi targeting *DcCathB* and *DcCathL* genes in *D. citri*. The RNAi were constructed and delivered via modified CTV vectors. Our results showed a significant downregulation of both *Cathepsin* genes in ACP adults. Additionally, we examined the effects of these RNAi on ACP female reproduction, and our results indicated coincident downregulation of the *Vitellogenin A1-like* gene, which was associated with significant reduction in ovarial development among *D. citri* females feeding on RNAi-treated plants infected with modified CTV vectors.

## 2 Materials and methods

### 2.1 Plants and insects

*Nicotiana benthamiana* plants were maintained in a growth-room under controlled conditions set at 22–24°C, 16:8 h photoperiod 60% relative humidity (RH). One year old *Citrus macrophylla* seedlings were maintained under controlled greenhouse conditions of 23 ± 3°C, 60 RH, and a 16:8 h (Light: Dark) photoperiod with a maximum photosynthetic radiation of 215 µmol s^-1^ m^-2^. Plants were watered twice per week and fertilized twice per month with an alternating schedule of a 24-8-16 NPK solution at 4 g L^-1^ (Miracle-Gro All Purpose Plant Food; Scotts Miracle-Gro Products, Marysville, OH) and a 6-4-6 (N–P–K) granular fertilizer at 1 g per pot (Expert gardener Gro Tec. Inc. Madison, GA).

A laboratory population of *D. citri* with known susceptibility to insecticides was reared in a greenhouse at the Citrus Research and Education Center, University of Florida, Lake Alfred, FL. This population originated from adults collected in 2000 from citrus in Polk City, Polk County, FL and has been reared without exposure to insecticides. The culture was maintained on sweet orange (*Citrus sinensis* Osbeck) in a greenhouse at approximately 27 °C, with 60% relative humidity, and a 14:10 (light: dark) photoperiod.

### 2.2 Citrus tristeza virus (CTV) based vectors

The infectious cDNA clone of Citrus tristeza virus (CTV isolate T36; GenBank accession no. AY170468) in the binary vector pCAMBIA-1380 was used for engineering all the constructs used in this study (Satyanarayana et al., 1999; Satyanarayana et al., 2001; Gowda et al., 2005; El-Mohtar and Dawson, 2014). To clone *Cathepsin B* and *Cathepsin L* fragments and generate the corresponding CTV vector, primers were designed based on *D. citri* genes (GenBank accession no. NM_001329180 and MN166228.1) (**Table 1**). The fragments corresponding to each *D. citri* gene were amplified using 2 µg of total RNA as template using the Super Script^®^III One-Step RT-PCR System with Platinum^®^Taq DNA Polymerase (Life Technologies Corp.), 100 nM of each primer (**Table 1**) in a final volume of 100 µL adjusted with nuclease-free water (Thermo fisher). The PCR products of each gene were digested with *PacI* and *StuI* restriction enzymes and ligated into their corresponding digested CTV vector. CTV binary plasmid vectors were screened by restriction digestion and confirmed by sanger sequencing (Psomagen, Inc, USA).

**Table 1:** Sequence of specific primers for the *DcCathL* and *DcCathB* genes of *D. citri*.

PCR primers Amplicon size (bp)		
M-1337 F M-1338 R	5’ TATAGGCCT TGCAAGTACGGTAACCAGGGATGCAAT 3’ 5’ TCCTTAATTAACCAGGTGGTGTTCCAGGAGTTTTTGA 3’	412
M-1339 F M- 1340 R	5- TCTAGGCCT CTGCAAAACTGTACCCTACTGGGTAA -3 5- TCG TTAATTAA TTCTCCCCACGGAGTATTTTGAATGT -3	390

Sequence of primers used for cloning based on the DcCathL (M-1337 Forward and M-1318 Reverse) and DcCathB (Forward M-1339 and Reverse M-1340) genes of D. citri.

### 2.3 Agroinfiltration and CTV virion isolation and inoculation to citrus

The CTV constructs dsRNA-DcCathB and dsRNA-DcCathL were used to transform *Agrobacterium tumefaciens* cells (EHA 105) and then, the transformant colonies were selected on LB plates containing the antibiotics Rifampicin (50 µg/ml) and Kanamycin (50 µg/ml). The transformant colonies were grown in LB liquid medium with Kanamycin (50 µg/ml), supplemented with 10 mM MES (pH 5.8) and 20 mM acetosyringone. This culture was used to prepare a agrobacteria cellular suspension containing the target constructs and infiltrated in the abaxial side of *N. benthamiana* leaves following the protocol described previously (Gowda et al., 2005; Ambrós et al., 2011). Systemic leaves from *N. benthamiana* that assessed positive for CTV by ELISA were harvested after 8 weeks post infiltration and used to isolate recombinant CTV virions by concentration over a sucrose cushion (Robertson et al., 2005). Thus, the solution containing the purified recombinant virions were used for bark-flap inoculation of *C. macrophylla* as described previously (Robertson et al., 2005).

### 2.4 Insect bioassays

Adult *D. citri* were harvested from the main culture described above and isolated as group of 50 females and 50 males in cages with four 1-year old *M. paniculata* plants for 10 days to allow egg laying. Then, adult insects were removed, and development of nymphs was tracked daily. On the day adults emerged, insects were separated by sex and color morph (only green/blue color morph insects were used in this study) and then transferred to 2-year-old *C. macrophylla* plants for 24 hours. On the second day after adult emergence, forty green/blue females and males per biological replicate were relocated onto 1-year old *C. macrophylla* seedlings (approximately two feet tall and stem 1 cm of diameter) colonized independently with CTV-*DcCathB* and -*DcCathL* RNAi vectors. The negative control used for experiments was *Citrus macrophylla* seedling infected with CTV-wt vector. For gene expression analysis, groups of 10 female and 10 *males D. citri* adults were collected 5 and 10 days after assays were initiated per biological replicate (N= 4 replicates). Similarly for analysis of ovary morphology, ten females per replicate were collected at day 10 and ovaries were dissected as previously described in Ibanez et al. (2019). In total four independent replicates were conducted per assay.

### 2.5 RNA extraction and cDNA synthesis

The total RNA for each pool of 10 insects per biological replicate was extracted after sample homogenization at room temperature with a plastic pestle using 500 µL of TRIzol reagent (Thermo Fisher scientific, MA) following the manufacturer’s instructions. Traces of genomic DNA were removed by DNase treatment using the TURBO DNA-free kit (Thermo Fisher scientific). The total RNA quantity and purity was evaluated on a Nanodrop Spectrophotometer (Thermo fisher Scientific), and the integrity of the RNA was visualized in 1.2% agarose gel electrophoresis. The synthesis of cDNA was performed using the Verso cDNA Synthesis kit (Thermo fisher Scientific). Each retro-transcription synthesis was conducted using five hundred ng of total RNA as template and adding anchored-Oligo (dT) primers, following manufacturer recommendations (Thermo Fisher scientific).

### 2.6 Gene expression analyses

Quantitative PCR (qPCR) reactions were performed using PowerUp SYBR Green Master Mix (Applied Biosystems). Each qPCR reaction contained 10 ng of cDNA as template, 300 nM of each primer (**Table 2**) and 1x of PowerUp SYBR Green Master Mix; in a final volume of 10 µL adjusted with nuclease-free water (Thermo fisher). The PCR program consisted of 2 min 50°C for UDG activation, 2 min 95°C for Dual-Lock DNA polymerase followed by 40 cycles at 95°C for 15 sec and 60°C for 1 min. Primers for each target gene were designed using Primer3 web (Untergasser et al., 2012). Each qPCR reaction was performed in duplicates with a negative control in each run using an Applied Biosystems ABI 7500 real-time PCR thermocycler (Applied Biosystems). The amplification specificity of each PCR product was monitored using the melting curve analysis in Sequence detection system (SDS) version 1.4.0.27 (Applied Biosystems) and visualizing the PCR products in a 2% agarose gel. The relative gene expression was estimated by the 2^-ΔΔCT^ method (Livak and Schmittgen, 2001), using *β-actin* as a reference gene (GenBank XM_026821238.1) as was previously described (Hajeri et al., 2014).

**Table 2:** Sequence of qPCR primers for *D. citri* genes.

qPCR primers Amplicon size (bp)		
*Dc CathB* F *Dc CathB* R	5-ATTGGCGATCTACGTTCCAC-3 5-GCCAGGTTGATGTGACATTG-3	95
*Dc CathL* F *Dc CathL* R	5-TGGCAGCTGTTTCATTTGAGG-3 5-TTCCTCAATGTCCGACTCGT-3	92
*Dc Vg A1-like* F *Dc VgA1-like* R	5-TATTGGCTGTTCTCCCCAAC-3 5-TGGTGACTTGAGAGCTGGTG-3	90
*Dc β-actin* F *Dc β-actin* R	5-TGTTCCAACCTTCCTTCCTG-3 5-GTGTTGGCGTACAGGTCCTT-3	109

Sequence of primers for analysis of gene expression of DcCathB (Dc CathB-F and Dc CathB-R), DcCathL (Dc CathL-F and Dc CathL-R), Vitellogenin A1-like (Dc Vg A1-like-F and Dc VgA1-like -R) by qPCR. Real-time expression analysis was normalized by the β-actin reference gene (Dc β-actin-F and Dc β-actin-R).

### 2.7 Statistical analysis

To examine the effects of each CTV construct in *D. citri*, one-way analysis of variance (ANOVA) with Tukey’s *post hoc* test was used to compare mean gene expression and numbers of mature oocytes between treatments. All statistical analyses were performed using RStudio environment (RStudio Team, 2015).

## 3 Results

### 3.1 CTV-based citrus plant-mediated RNAi suppressed expression of *DcCathB* and *DcCathL* in *D. citri* adult

**Figure 1:**
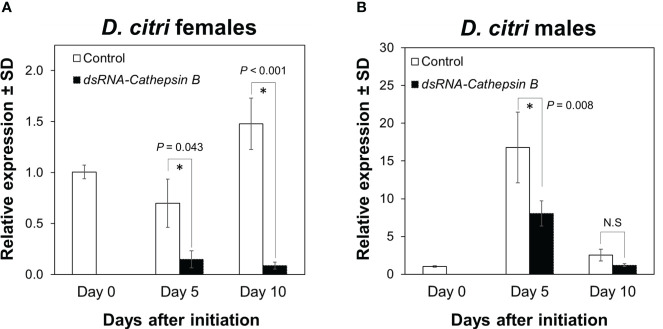
Expression of *Cathepsin B* in *Diaphorina citri* adults. The temporal pattern of *Cathepsin B* transcript relative expression in *D. citri* females **(A)** and males **(B)** was normalized to the expression value of the *β-actin* transcript. Data represent means ± standard deviations (SD) of 4 independent replicates. Asterisks indicate statistical differences between gene expression in psyllids fed on dsRNA-*DCcathB* or control plants using one-way ANOVA with Tukey’s *post hoc* test. N.S denotes no significance.

Expression of the *DcCathB* transcript in female *D. citri* was significantly reduced (F = 19.325, df = 4, P < 0.001) after 5 and 10 days of feeding on plants colonized with CTV RNAi vectors targeting *DcCathB*. *DcCathB* expression was reduced by 79% at day 5 (P = 0.043), and 95% after 10 days (P < 0.001) of feeding on plants expressing dsRNA- *DcCathB*, compared to control females at the same time points (**Figure 1A**). In *D. citri* males, the *DcCathB* transcript was also significantly reduced (F = 15.618, df = 4, P < 0.001). Pairwise comparisons at day 5 showed a 52% reduction in *DcCathB* expression (P = 0.008), while at day 10, there was no significant difference (P = 0.98) compared to male controls at the same time points (**Figure 1B**).

**Figure 2:**
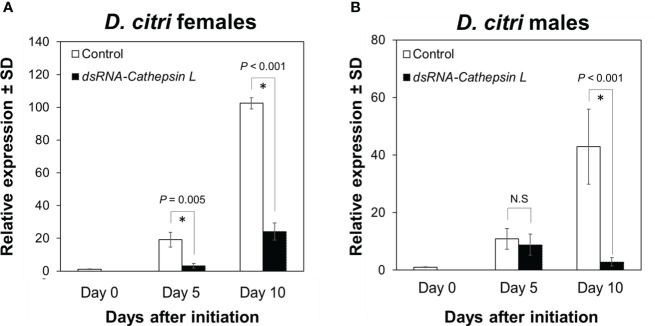
Expression of *Cathepsin L* in *Diaphorina citri* adults. The temporal pattern of *Cathepsin L* transcript relative expression in *D. citri* females **(A)** and males **(B)** was normalized to the expression value of the *β-actin* transcript. Data represent means ± standard deviations (SD) of 4 independent replicates. Asterisks indicate statistical differences between gene expression in psyllids fed on dsRNA- *DcCathL* or control plants using one-way ANOVA with Tukey’s *post hoc* test. N.S denotes no significance.

Similarly, exposure of female and male *D. citri* for 5 and 10 days of feeding on plants colonized by CTV RNAi vectors targeting *DcCathL* led to significantly reduced expression of the *DcCathL* transcript (F = 181.585, df = 4, P < 0.001). *DcCathL* expression was significantly reduced by 83% (P = 0.005) and 77% (P < 0.001) at 5 and 10 days after feeding was initiated, respectively, compared with control females at the same time points (**Figure 2A**). In *D. citri* males, expression of the DcCathL transcript was also significantly reduced (F = 9.510, df = 4, P < 0.001). Pairwise comparisons between controls and RNAi treatments at day 5 showed no significant change in *DcCathL* expression (P = 0.43), while at day 10, the *DcCathL* transcript was significantly reduced (P = 0.0005) by 96% compared to male controls at the same time point (**Figure 2B**).

### 3.2 CTV-based citrus plant-mediated RNAi (*DCcathB* and *DCcathL*) suppresses expression of *Vitellogenin A1-like* and reduces ovary maturation in *D. citri* females

**Figure 3:**
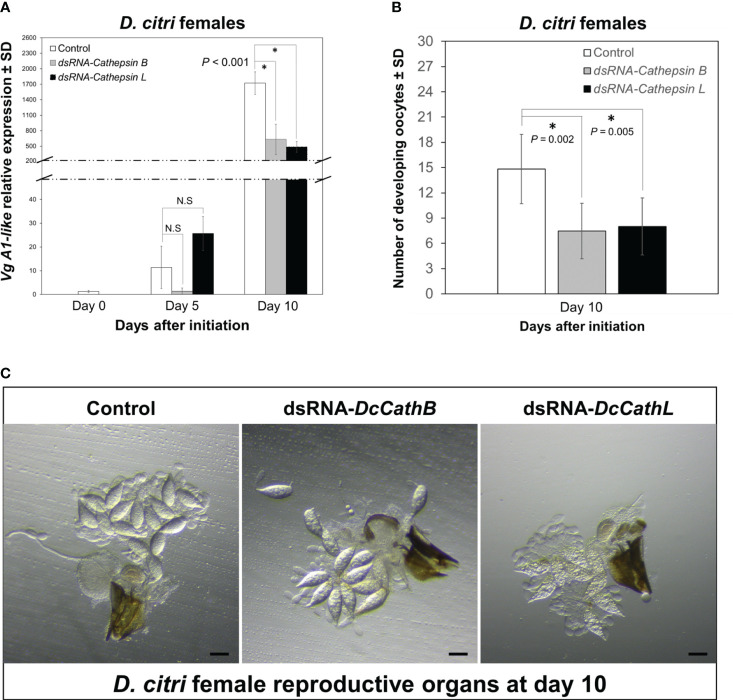
Effects of CTV-based RNAi (*DCcathB* and *DCcathL*) via expression in citrus plants on *D. citri* female reproductive development. **(A)** Temporal pattern of *Vg1-like* transcript relative expression levels in *D. citri* females normalized to the expression value of the *β-actin* transcript. Data represent means ± standard deviations (SD) of 4 independent replicates. Asterisks indicate statistical differences between gene expression in psyllids fed on plants expressing CTV-based dsRNAs (*DcCathB* and *DcCathL*) or control plants using one-way ANOVA with Tukey’s *post hoc* test. N.S denotes no significance. **(B)** Number of developing oocytes detected in *D citri* females after 10 days of challenge to plants expressing CTV-based dsRNAs or control plants. Data represents means ± SD (standard deviation) of at least thirty dissected females per group. **(C)** Representative images of ovaries from *D. citri* females. Scale bar is equal to 100 µm.

To evaluate if knockdown of *Cathepsin* expression interferes with *D. citri* reproduction, the expression of *Vg A1-like* and number of developing oocytes in females feeding on citrus plants colonized with CTV-based RNAi vectors independently targeting *DcCathB* or *DcCathL* were analyzed using pairwise comparisons. The relative expression of *Vg A1-like* was significantly (F = 51.542, df = 6, P < 0.001) downregulated in both treatments as compared with expression observed in controls after 10, but not 5 (P > 0.05), days of feeding (**Figure 3A**). The significant difference in *Vg A1-like* expression at day 10 prompted us to dissect the reproductive organs of female *D. citri* feeding on CTV-based RNAi colonized citrus (*DcCathB* or *DcCathL*) and compare them versus those feeding on control plants (**Figures 3B, C**). Significantly more developing oocytes were counted in the ovaries of control *D. citri* females than those fed on plants colonized with CTV-RNAi vectors independently targeting *DcCathB* (P = 0.002) or *DcCathL* (P = 0.005) (**Figure 3B**).

## 4 Discussion

Cathepsin B and cathepsin L enzymes in insects are associated with various biological processes including protein turnover, embryogenesis, mobilization, degradation of yolk proteins (Medina et al., 1988; Izumi et al., 1994; Cho et al., 1999; Zhao et al., 2002), metamorphosis (Takahashi et al., 1993; Shiba et al., 2001; Lee et al., 2009), and programmed cell death (Yang et al., 2020). We hypothesized that cathepsin enzymes should be useful targets for RNAi given their broad importance for *D. citri* development. Given the current lack of therapeutic treatments targeting the *C*Las pathogen, emphasis has been placed on reducing populations of the *D. citri* vector (Hajeri et al., 2014).

RNAi has been demonstrated on target insects for cathepsin genes using transgenic plants (Huang et al., 2006; Galdeano et al., 2017). In the present investigation, we utilized CTV-RNAi to target the cathepsin genes in *D. citri*. Delivery of dsRNA in citrus plants against a phloem feeding insect, such as *D. citri*, using CTV-based vectors offers advantages over the traditional transgenic approach in citrus. Engineering the virus is relatively easier and faster than producing transgenic plants. The engineered virus could colonize the mother plants from which budwood is used to generate citrus trees for establishing new grooves. Further, the CTV virus vector could be graft transmitted to current plants at any stage, including fully grown plants in the ground, obviating the need to remove the current generation of plants. However, despite the stability of CTV RNAi vectors for many years, at some stage, the engineered virus will recombine back into the wild type of the virus (El-Mohtar and Dawson, 2014). During this time, we expect that ongoing research may provide the ultimate solution for HLB via engineering a resistant/tolerant plant via biotechnology or traditional breeding.

*DcCathB* gene expression occurs in the *D. citri* gut of both nymph and adult stages, suggesting a possible role in digestion (Ferrara et al., 2015). Additionally, downregulation of *DcCathB* occurs in the presence of the *C*Las bacterium of both nymphs and adults, suggesting a possible role in immune response (Vyas et al., 2015). A different pattern of *DcCathL* expression has been reported compared to *DcCathB*, with expression observed in the head, thorax, and gut tissues of *D. citri*, suggesting function in more processes than digestion. Greater expression of the *DcCathL* gene in eggs than nymph and adult stages also suggests a role of in embryonic development (Ferrara et al., 2020). Additionally, *DcCathL* has been implicated in the innate immune response, like *DcCathB* (Yu et al., 2019). The preponderance of evidence collected to date would suggest that both *DcCathB* and *DcCathL* play roles associated with development in *D. citri*.

Our results provide evidence that *DcCathB* and *DcCathL* are associated with female reproductive development in *D. citri* (**Figure 3**). Following knockdown of either *DcCathB* (**Figure 1**) or *DcCathL* (**Figure 2**), oocyte development was significantly compromised (**Figure 3B**) and resulted in an abnormal ovary phenotype (**Figure 3C**). Furthermore, maturation of ovaries was delayed, which appeared related to interference with vitellogenesis (**Figure 3**). It is possible cathepsin gene regulation plays a role in vitellogenesis at some stage given the observed downregulation of the *Vitellogenin A1-like* gene coincident with downregulation of *DcCathB* or *DcCathL* (**Figure 3**).

**Figure 4:**
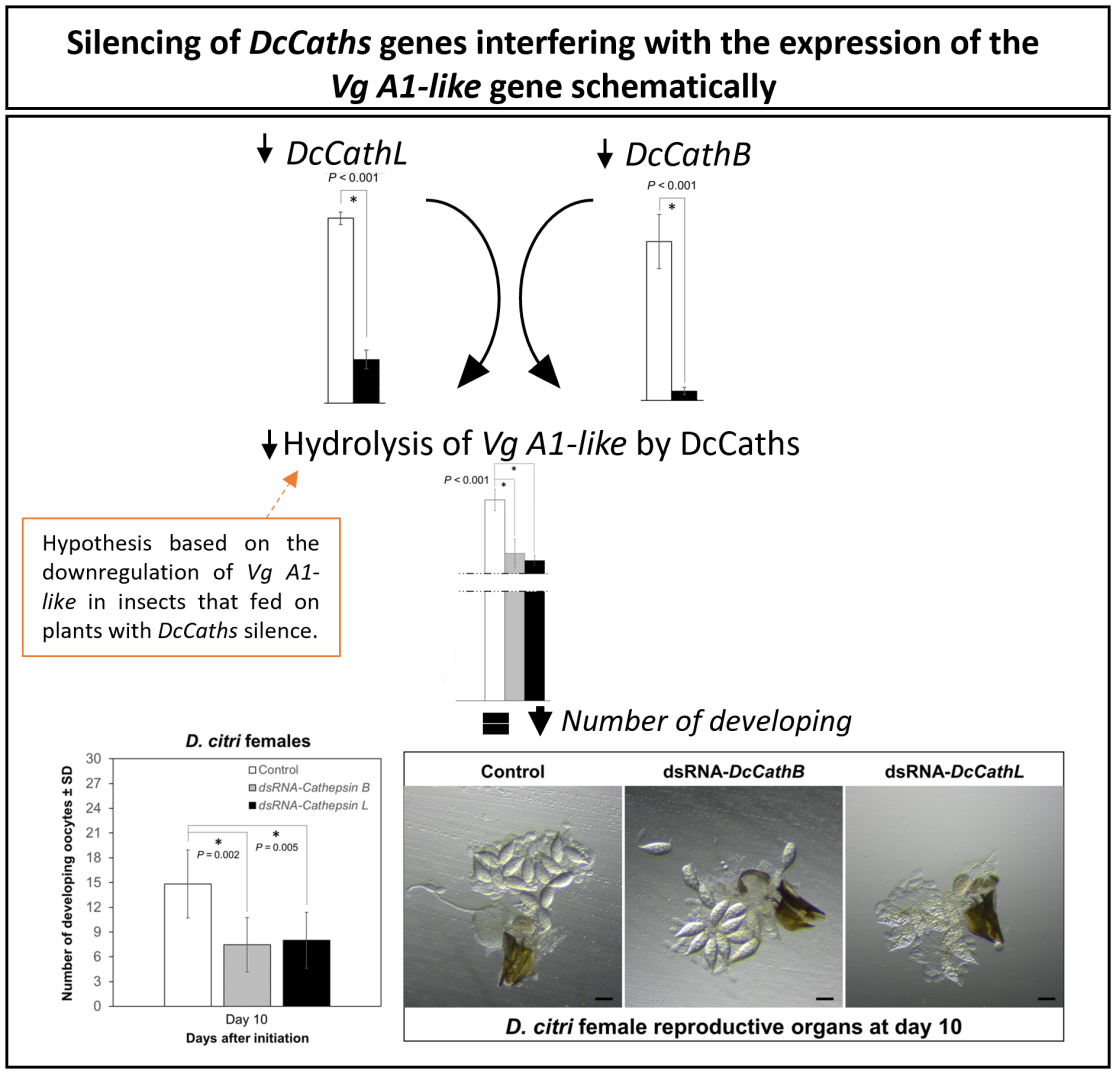
Hypothesis of *Vitellogenin* (*Vg*) and *Cathepsins B* and *L-like* (*DcCathB* and *DcCathL*) relationship. *Vitellogenin* (*Vg*) downregulated regarding *DcCathB* and *DcCathL* gene silencing resulting in decreased number of developing oocytes as well an ovarian development compromised.

Vitellogenesis is crucial for insect embryonic development and egg production (Wu et al., 2021), and alterations in *Vitellogenin* (*Vg*) and associated genes can significantly interfere with normal development. For example, interference with *Vg* and the Vitellogenin receptor (*VgR*) hinder vitellogenin accumulation and resultant egg maturation (Ibanez et al., 2017; Peng et al., 2020). Similarly, in locusts, reduced expression of Vg gene resulted in arrested oocyte maturation and impaired egg production (Guo et al., 2014; Wu et al., 2016; Wu et al., 2018). In *D. citri*, knockdown of *Vitellogenin-4 like* (*Vg-4 like*) and *VgR* genes decreases the number of mature eggs in the ovary and causes phenotypic abnormalities in eggs and nymphs (Li et al., 2022). Also, RNAi knockdown of the *VgR* gene in *Cadra cautella* resulted in low fecundity and fertility (Husain et al., 2020). Cathepsin L (*Mn-CTS L1*) has been reported to play an important role in the ovarian development of the oriental river prawn, *Macrobrachium nipponense*. Interestingly, it was hypothesized that *Mn-CTS L1* silencing can effectively inhibit Vg hydrolysis, thereby reducing the availability of energy required for ovarian development (Zhu et al., 2021). Cathepsin B has been shown to play a role in embryogenesis and egg development in the hard tick, *Haemaphysalis longicornis* (Zhang et al., 2019). Accordingly, we could observe a downregulation of *Vg A1-like* in those insects that fed on plants expressing dsRNA-*DcCaths* (**Figure 4**). In addition, Cathepsins have been related to embryogenesis in insects as well the *Vg* hydrolysis (Moura et al., 2015; Zhang et al., 2019), for instance resulting in a reduction in developing oocytes and compromising ovarian maturation (**Figure 3**).

However, how *cathepsin* and *vitellogenin* genes interact and why knockdown of *DcCathB* or *DcCathL* both appeared to coincidentally reduce vitellogenesis remain open questions.

Novel methodologies such as RNAi must be considered to mitigate economic and environmental damage of pathogen vectors (Zhang et al., 2013). We demonstrate here the silencing of cathepsin genes in *D. citri* by delivery of RNAi inducers via an engineered CTV vector colonizing citrus phloem tissue. Given that CTV accumulates and produces large amounts of dsRNA in the phloem, which is the sole source of nutrition for developing *D. citri* (Hajeri et al., 2014), use of CTV-based RNAi vectors is being explored as possible management tools for this phytopathogen vector. Our results indicate that reducing cathepsin gene expression causes visible phenotypic changes in *D. citri*, delaying and reducing ovarial development which appears related to a downregulation of *vitellogenin* expression. It will be important to determine whether reduced ovarial development is associated with changes to the fecundity and fertility of *D citri* (Bueno et al., 2020; Koji et al., 2020). We speculate that decreased ovarial development caused by silencing of cathepsin genes may result in reduced population growth of *D. citri*. This hypothesis will require further testing. The remarkable stability of the CTV vector, its effectiveness in delivering dsRNA into the plant phloem, and relatively fast activity further support its use for validation of biological targets and development into practical therapeutic tools. Our findings also indicate that Cathepsin B and L-like are promising target sites that should be further explored as a means of managing the *D. citri* vector. This research represents an initial step that will require scaling up to population-wide treatment of psyllids under authentic field conditions. Further biological testing of the consequences of *cathepsin* silencing in *D. citri* are needed to determine potential practical utility for population suppression of this phytopathogen vector, including effects on female fecundity and fertility.

## Data availability statement

The datasets presented in this study can be found in online repositories. The names of the repository/repositories and accession number(s) can be found in the article/supplementary material.

## Author contributions

AS-C, FI-C, CEM, CR, WD and LS contributed to conception and design of the study. AS-C, SVR and FI-C wrote sections of the manuscript. All authors contributed to the article and approved the submitted version.
